# γδ T Lymphocytes as a Double-Edged Sword—State of the Art in Gynecological Diseases

**DOI:** 10.3390/ijms232314797

**Published:** 2022-11-26

**Authors:** Anna Pawłowska, Yelizaveta Natochina, Witold Zardzewiały, Wiktoria Skiba, Karolina Włodarczyk, Aleksandra Maciejczyk, Dorota Suszczyk, Iwona Wertel

**Affiliations:** 1 Independent Laboratory of Cancer Diagnostics and Immunology, Medical University of Lublin, Chodźki 4a, 20-093 Lublin, Poland; 2 Students’ Scientific Association, Independent Laboratory of Cancer Diagnostics and Immunology, Medical University of Lublin, Chodźki 4a, 20-093 Lublin, Poland; 3 Department of Functional Anatomy and Cytobiology, Institute of Biological Sciences, Maria Curie-Sklodowska University, Akademicka 19, 20-033 Lublin, Poland

**Keywords:** gamma-delta (γδ) T cells, ovarian cancer, tumor microenvironment (TME)

## Abstract

Human gamma-delta (γδ) T cells are a heterogeneous cell population that bridges the gap between innate and acquired immunity. They are involved in a variety of immunological processes, including tumor escape mechanisms. However, by being prolific cytokine producers, these lymphocytes also participate in antitumor cytotoxicity. Which one of the two possibilities takes place depends on the tumor microenvironment (TME) and the subpopulation of γδ T lymphocytes. The aim of this paper is to summarize existing knowledge about the phenotype and dual role of γδ T cells in cancers, including ovarian cancer (OC). OC is the third most common gynecological cancer and the most lethal gynecological malignancy. Anticancer immunity in OC is modulated by the TME, including by immunosuppressive cells, cytokines, and soluble factors. Immune cells are exposed in the TME to many signals that determine their immunophenotype and can manipulate their functions. The significance of γδ T cells in the pathophysiology of OC is enigmatic and remains to be investigated.

## 1. Introduction—Subpopulations of Gamma Delta (γδ) T Lymphocytes

Gamma-delta (γδ) T cells are part of the innate and acquired immune systems, accounting for 0.5–5% of all peripheral blood (PB) lymphocytes [1,2,3,4]. They predominantly exist in mucosal tissues, such as the skin, lungs, small intestine, and the female reproductive organs—e.g., the uterus and the ovary [5,6]. 

γδ T lymphocytes do not express CD4 and CD8 molecules and are described as double-negative (DN, CD4^−^ CD8^−^) lymphocytes [7]. Unlike conventional αβ T cells, they can recognize non-MHC antigens. They serve the important function of identifying heat shock proteins and super antigens [5]. γδ T cells interact via their T cell receptors (TCR γδ) and natural killer (NK) cell receptors, such as natural killer group 2D (NKG2D) receptors and natural killer cell receptors (NKRs) without Major Histocompatibility Complex (MHC) restriction [2,3]. NKRs bind to surface proteins associated with disease or stress conditions on malignant cells [8]. This subset of cells has the ability to activate quickly, being the body’s first line of defense against pathogens and playing a central role in anticancer immunity [1]. γδ T cells can generate immune memory [1,4]. Regarding the expression of TCRγ chains or TCRδ chains, human γδ T cells are divided into the subpopulations of Vδ1, Vδ2, and Vδ3 T cells based on the delta chain of the T cell receptor (TCR). Moreover, γδ T cells can be separated into two subsets, Vδ2-positive and Vδ2-negative, because of the expression of the Vδ chain. The major subsets of circulating blood γδ T cells are Vδ2-positive, interconnected with the Vγ9 chain to the Vγ9Vδ2 form of lymphocytes as the major peripheral blood subpopulation [3,9]. It should be stressed that Vδ1 and Vγ9Vδ2 T cells are the two main subsets of γδ T cells in human tissue and PB [2].

Vγ9Vδ2 T cells are activated mostly by non-protein pyrophosphate metabolites called phosphoantigens (pAgs), which are mevalonate products in the isoprenoid pathway or the non-mevalonate Rohmer pathway [6]. The subsets of Vγ9Vδ2 T cells have strong antitumor activity, explaining their wide usage in clinical settings. Numerous clinical studies have used aminobisphosphonates (e.g., zoledronate and pamidronate) to inhibit farnesyl pyrophosphate synthase in the mevalonate pathway to promote the accumulation of isopentenyl pyrophosphate (IPP) in cells, or synthetic phosphoantigen analogues, such as bromohydrin pyrophosphate (BrHPP) and 2-methyl-3-butenyl-1-pyrophosphate (2M3B1PP), to activate Vγ9Vδ2^+^ T cells in patients with malignant tumors [10].

Unlike Vδ2-positive T cells, Vδ1 and Vδ3 are more common in tissue than in peripheral blood lymphocytes. The majority of Vδ1 T cells are found in the mucous membrane, proliferating mostly in peripheral tissues, including solid tumors [8]. Under stress, T cells secrete factors that penetrate damaged, infected, or malignant cells, inducing inflammation. It is important to note that the role of Vδ1 T cells in cancer immunity is not well-established [6]. However, one interesting quality of these subsets is their CCL2-mediated migration into tumors, which makes them a potential tool for clinical manipulation in cancer immunotherapy [2].

Vδ3 T cells are primarily located in the liver and intestines and are implicated in response to herpes virus infections, cytomegalovirus and Epstein–Barr virus [9]. The role of Vδ3 T cells in cancer immunity has not been studied in depth [6].

The purpose of this work is to summarize existing knowledge about the phenotype and dual role of γδ T cells in gynecological diseases, including ovarian cancer (OC).

## 2. Variations in the Phenotype and Function of γδ T Lymphocytes in Human Cancers

**Figure 1:**
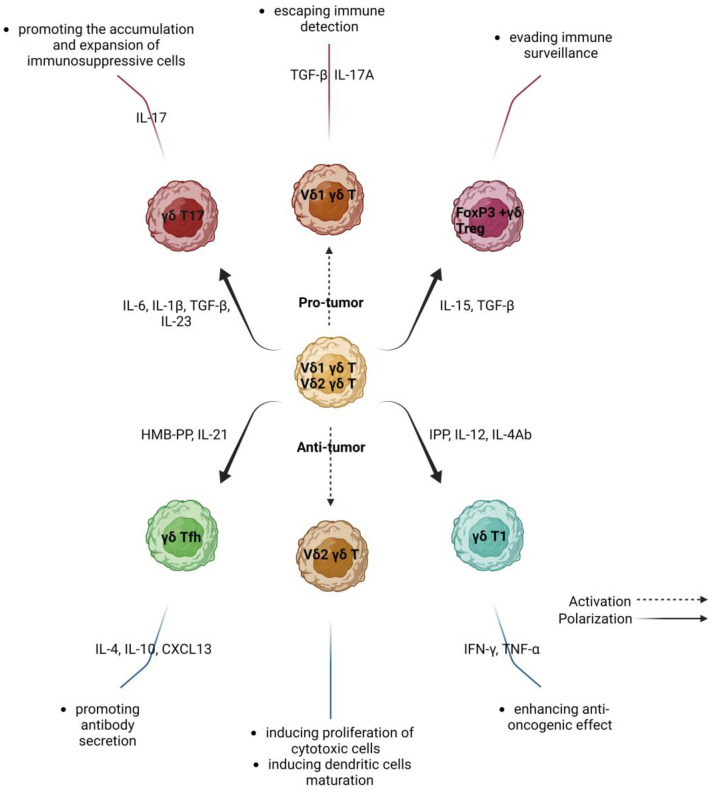
Subpopulations of gamma delta T lymphocytes and their activity in cancers.

Human γδ T cells are a heterogeneous cell population that bridges the gap between innate and acquired immunity. They are involved in a variety of immunological processes, including tumor escape mechanisms. However, by being prolific cytokine producers, these lymphocytes also participate in antitumor cytotoxicity. Which one of the two possibilities takes place depends on the tumor microenvironment (TME) and the subpopulation of γδ T lymphocytes (Figure 1.). Interestingly, γδ T lymphocytes can switch phenotypes in response to TME signals [10,11]. Immune system cells are exposed in the TME to many signals that determine their immunophenotype and can modulate their functions [12]. The latest findings in tumor biology suggest that most cancers are immunogenic. Hence, the tumor microenvironment appears to be a promising target for potential treatments [13,14].

Depending on the microenvironment, γδ T cells perform antagonistic roles through the secretion of various cytokines. Resting γδ T cells can differentiate into protumor subgroups—FoxP3^+^ γδ Treg, γδ T17, Vδ1 γδ T cells—and antitumor subgroups—γδ Tfh, Vδ2 γδ T, γδ T1 cells.

## 3. γδ T Lymphocytes in Cancer—Friends or Foes?

### 3.1. Protumor Activity of γδ T Lymphocytes

Cancer research has highlighted the role of γδ T lymphocytes as the most significant favorable immune prognostic factor associated with overall survival (OS) outcomes across many malignant tumors [15]. Increased Vδ1 infiltration into tumor tissue has recently been shown in multiple solid cancers, including colorectal cancer, melanoma, and non-small cell lung cancer, as well as in several studies involving ovarian cancer [8,16,17]. Weimer et al. [8] found the presence and accumulation of Vδ1 T cells in ascites, and among tumor-infiltrating cells (TILs) from ovarian cancer patients. It is worth stressing that malignant ascites acts as a transporter facilitating the spread of highly carcinogenic tumor cells (TCs) to pelvic and peritoneal cavities in OC patients [18]. In a study by Weimer et al. [8], γδ T lymphocytes accounted for about 3% of total CD3^+^ T cells in OC patients. This percentage was significantly higher than for Vδ2 T cells. Vδ1 T lymphocytes represented the dominant subsets of γδ T cells in ascites and TILs, whereas Vδ2 T cells constituted the majority of peripheral blood T lymphocytes in both OC patients and controls [8]. Foord et al. [19] showed similar distributions of γδ T lymphocytes in PB, ascites, and TILs in OC patients. In another study, Chen et al. [16] found significantly higher percentages of γδ T cells and Vδ1 T cells in OC tissues compared to non-malignant and normal ovarian tissues. The study showed a relationship between increased Vδ1 infiltration in OC tissues and the progression of OC—e.g., a more advanced clinical International Federation of Gynecology and Obstetrics (FIGO) stage and lymph node metastasis. These findings point to the critical role of this population in OC progression and invasiveness [16]. In addition, higher proportions of γδ T cells correlated with a shorter disease-free intermission in patients with advanced ovarian cancer [20]. Furthermore, the increased ratio of Vδ1/Vδ2 T cells seems to have prognostic significance in OC [17].

In one study on OC, Weimer et al. [8] provided a phenotypic characterization of matched γδ T lymphocytes in PB, ascites, and TILs in OC patients. They found that Vδ1 cells in ascites showed an increased number of cells carrying a terminally differentiated (TEMRA) phenotype, with an aberrant subpopulation of CD27-CD45RA (high) Vδ1 T cells. In contrast, the increased CD27-CD45RA effector memory (EM) differentiation cells were dominant in TILs. Interestingly, the authors noted differences in the expression of individual co-regulatory receptors (CRRs) on Vδ1 cells in distinct OC-related compartments. In peripheral blood, Vδ1 T cells exhibited an increased frequency of negative immune checkpoints (ICPs), such as T cell immunoglobulin and ITIM domain (TIGIT; also called Vstm3, WUCAM, VSIG9), T cell immunoglobulin and mucin domain-containing molecule-3 (TIM-3), and Ox40 on the T cell’s surface. Vδ1T cells in ascites showed an increased frequency of TIGIT^+^ and TIM-3^+^ cells, whereas TILs had higher frequencies of PD-1^+^, CD39^+^, and Ox40^+^ cells in comparison to Vδ1 cells from PB of controls. In contrast, all the γδ T cells showed a lower percentage of CD73-positive cells. Finally, despite the correlation between immune checkpoint expression and differentiation stage, increased co-expression of PD-1, TIM-3, and CD39 with TIGIT was detected in all Vδ1 γδ T cells across the OC group. These findings indicate an increased state of exhaustion in Vδ1 T cells in OC [16]. Interestingly, γδ T cells sorted from OC tissues exhibited lower cytotoxic activity against ovarian cancer cells. γδ T cells cocultured with OC tissue supernatants effectively inhibited the proliferation of naïve CD4^+^ T cells [16].

In other studies, immunosuppressive or tumor-promoting capabilities have also been described for γδ T cells, especially via the secretion of interleukin-17 (IL-17). Chen et al. [16] reported that in ovarian cancer patients, γδ T cells produce increased levels of IL-17A. Conversely, interferon-gamma (IFN-γ)—as an antitumor factor—was at a significantly low level [16,17]. Recent reports have suggested that IL-17A could induce immunosuppression and facilitate tumor progression. Higher expression of IL-17A was demonstrated in some cancers, including cervical cancer, breast cancer, non-small cell lung cancer, pancreatic cancer, and hepatocellular carcinoma [16]. Furthermore, it has been proven that Th17 γδ T cells may stimulate angiogenesis by producing angiogenic factors such as angiopoietin 2 (ANG-2) and vascular endothelial growth factor 2 (VEGF) [21]. Meanwhile, IL-17A could polarize inflammatory macrophages and recruit myeloid-derived suppressor cells (MDSCs) to the TME [16]. The recruitment of immunosuppressive MDSCs could also be mediated via the secretion of granulocyte-macrophage colony-stimulating factor (GM-CSF), interleukin-8 (IL-8), and tumor necrosis factor α (TNF-α) [22]. Furthermore, there is evidence that a higher percentage of Vδ1 T cells corresponds with immunosuppressive functions such as blocking dendritic cell (DC) maturation, limiting naive T cell proliferation, and suppressing the immune response of conventional αβ T cells [23]. Moreover, γδ T cells can negatively regulate αβ T cells’ response by increasing the expression of negative ICPs. The persistent chronic inflammation related to cancers and the prolonged stimulation of γδ T cells in the TME could trigger their immune exhaustion. It was demonstrated that tumor-infiltrating γδ T cells with higher expression of programmed cell death ligand 1 (PD-L1) and Galectin-9 (Gal-9) could inhibit the effector activity of conventional αβ T cells [10,24]. Moreover, the ligation of the CTLA-4 receptor on activated Vδ2^+^ γδ T cells with its ligand CD86 leads to the anergy of T cells and their elimination via apoptosis [25].

Ma et al. [26] claim that the level of γδ T cells in breast cancer correlates with a lower likelihood of survival and relapse. Liu et al. demonstrated that there is a positive relationship between ATPase Secretory Pathway Ca2^+^ Transporting 2 (ATP2C2) and Tfh cells in patients with BRCA-positive breast cancer [27]. There is evidence that patients with greater ATP2C2 levels have a shorter overall survival time. Moreover, Dang et al. [28] and Liu et al. [27] reported that Tfh cells in BRCA-positive breast cancer produce CXCL13 chemokine, which stimulates the infiltration of Tregs in the tumor tissue and limits the cytotoxic antitumor activity of the immune system [29]. Moreover, it has been proven that γδ Treg FOXP3^+^ cells inhibit the cytotoxic activity and proliferation of immune cells [11]. Interestingly, Tregs have been observed to accumulate in advanced stages of OC and considered a negative prognostic factor in patients with OC [30].

In colorectal cancer, tumor-infiltrating Vδ1 T cells were found to produce IL-17 and increase the presence of MDSCs, thus stimulating immunosuppression [23]. Their inherence correlated with the severity of the disease and cancer progression [17,31]. It has been suggested that an imbalance between Vδ1 T cells and Vδ2 T cells in favor of the former could conduce to rectal cancer development. Substantially more γδ T cells infiltrate colorectal cancer with BRAF or TP53 mutation. The same applies to tumors with proficient mismatch repair compared to deficient mismatch repair mutations [32].

Research on human gallbladder cancer has shown that the subpopulation of γδT17 cells promotes cancer progression by stimulating angiogenesis. The presence of this subpopulation is associated with poor survival rates [33].

In acute myeloid leukemia, it has been noted that the higher prevalence of γδ T cell immunoglobulin and immunoreceptor tyrosine-based inhibitory motif domain TIGIT^+^CD226- may correspond with a poorer prognosis [34]. Moreover, increased tumor infiltration by γδ T cells has been implicated in the increased likelihood of metastasis [6].

### 3.2. Anti-Tumor Activity of γδ T Lymphocytes

Active γδ T cells also show a strong cytotoxic effect against tumor cells in the TME [35]. They can produce INF-γ, as well as granzymes B and perforins, by activating TCR receptor and NKG2D receptor signaling on NK cells. NKG2D-expressing Vδ1^+^γδ T cells can be activated by stress-induced MHC class I chain-related antigens A and B (MICA/MICB) and UL16-binding proteins (ULBP16), which are upregulated in tumor cells contrary to healthy cells [10]. There is research showing that isopentenyl pyrophosphate stimulates higher expression of CD137 (4-1BBL) on γδ2^+^ T cells, which can lead to enhanced NKG2D expression after engagement with CD137-positive NK cells, and augment their cytotoxic activity against malignant cells [10]. Furthermore, some studies suggest that γδ T lymphocytes could also kill malignant cells through Fas/FasL pathway and antibody-dependent cell-mediated cytotoxicity (ADCC) [6,36].

Interestingly, there is research to suggest that γδ T lymphocytes may have features and functions similar to those of Th1, Th2, Th17, and regulatory T cells (Tregs). γδ T cells produce IFN-γ (such as Th1), IL-4 and IL-10 (such as Th2), and Il-17 (such as Th17). They also exhibit phenotypic similarity to Tregs, as they are involved in the regulation of immune processes. Some studies have reported that γδ T cells could support B cell activity, which is related to the production of IL-4 [37]. There is research evidence that activated Vγ9Vδ2^+^ T cells interact—directly or indirectly—with a range of immune cells, including DCs, monocytes/macrophages, αβ T cells, NK cells, and neutrophils. These cells have also been shown to influence immune response outcomes. The mechanisms underlying γδ T-cell unique immune-modulation activity are described in a recent review by Chan et al., which is a highly informative reference source on the subject [10].

The potential use of anti-cancer properties of γδ T cells awakens interest among the scientific community and clinicians [27]. It has been shown that γδ T cells have a natural tropism for the TME and that the presence of γδ tumor-infiltrating T cells in the TME correlates with a better prognosis for cancer patients. This has been proven by genomic data evaluation in over 18,000 human cancers [10,15]. For example, in melanoma patients, a higher percentage of tumor-infiltrating γδ T cells correlates with a longer progression-free survival (PFS) time [38]. Increased levels of Vδ2 T cells in melanoma were found to correlate with an early stage of disease and the absence of metastasis, and had a positive prognostic value [39]. There is also evidence to argue that Vγ9Vδ2 T cells are cytotoxic to renal cancer cells. One study found that γδ T cells selectively recognized renal cancer cells, distinguishing them from renal cells due to the presence of NKG2D on the surface of cancer cells [40]. The lytic abilities of the said cells were increased, especially after immunological enhancement. Strikingly, the use of donor γδ T cells was found to result in a higher treatment efficacy by increasing overall survival rates in acute myeloid leukemia, acute lymphoblastic leukemia, and chronic lymphocytic leukemia. Unfortunately, autologous γδ T cells did not produce the expected response [41]. There is research showing that Vδ1 and Vδ2 T cells present cytotoxic activity against endometrial carcinoma cell lines (KLE, Ishikawa, RL95-2). One study found that the level of Vδ1 T cells in peripheral blood was significantly reduced in patients with endometrial cancer. No similar relationship was noted for Vδ2 T cells. The study showed that tyrosine kinase EphA2 led to a relevant reduction in the lytic capacity of tumor cells by Vδ1 γδ T cells. Increasing EphA2 activity on cancer cells could create an opportunity for utilizing this property, not only in endometrial cancer. Moreover, the study noted that Vδ1 T cell mediated killing was substantially reduced in RL-95 cell EPHA2 knockout [4].

## 4. γδ T Lymphocytes in Autoimmune Diseases

It should be emphasized that tissue damage results in an increased number of γδ T cells, contributing to the acceleration of autoimmune pathologies mainly via the production of IL-17, TNF-α and IFN-γ [42,43,44,45,46,47,48,49]. Despite the main source of IL-17A being Th17 CD4^+^ αβ T cells, γδ T cells also contribute to IL-17A production in autoimmune diseases. Interestingly, IL-17-positive γδ T cells can expedite the development of autoimmune diseases by inhibition of Treg activity [43,50,51,52]. The T cell subset plays an important role in autoimmune diseases such as rheumatoid arthritis (RA), systemic lupus erythematosus (SLE), multiple sclerosis (MS), and endometriosis by having the capacity to present antigens, produce proinflammatory cytokines, enhance the release of antibodies, as well as through immunomodulatory activity and interactions with Tregs. However, data on the role of γδ T cells and their subtypes in the pathogenesis of autoimmune diseases are still limited.

For example, a stronger infiltration of γδ T cells is observed at the early stages of multiple sclerosis lesions and the subpopulation may comprise up to 20–30% of total T cells. Similarly, γδ T cells play a crucial role in lupus pathogenesis, and their number is significantly increased in previously untreated patients. Immunosuppressive therapy in patients with active lupus causes the normalization of γδ T cell percentage [45].

γδ T cells, equally with CD4^+^ T cells, play a complementary role in the production of cytokines in Behçet’s disease. The role of γδ T cells has been described as providing a link between innate and adaptive responses. Abbasova et al. showed that γδ T cells mainly produce IFN-γ, but they are not a source of IL-17A or IL-22. In an active disease, IL-17A comes mainly from CD4^+^ T cells. The study also indicates that γδ T cells are involved in the induction of inflammatory traits in Behçet’s disease [53,54].

Moreover, in autoimmune liver diseases, i.e., primary sclerosing cholangitis, autoimmune hepatitis, and primary biliary cirrhosis, the percentage of γδ T cells, including the Vδ1^+^, Vδ2^+^, and Vδ3^+^ subtypes, is increased in both liver and peripheral blood. These findings support the significance of this subset in autoimmune pathologies [43].

Furthermore, immunoregulatory γδ T cells can suppress the functions of dendritic cells and CD4-positive T cells. One explanation is that γδ T cells exert a stronger suppressing activity towards CD4^+^ T cell proliferation in comparison to CD4^+^ regulatory T cells. However, there is some discrepancy between the activity of proper subtypes of γδ T cells, and they are associated with diverse pathologies. The Vδ1 subset of γδ T cells is more inhibitive and involves higher TGF-β secretion compared to Vδ2 T cells [55,56,57].

**Figure 2:**
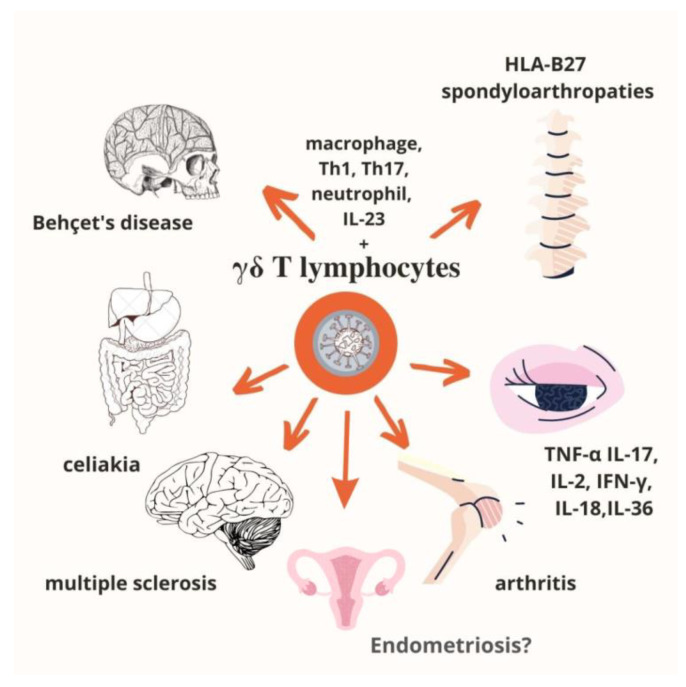
Autoimmune diseases associated with γδ T lymphocytes [35].

The regulatory activity of γδ T cells is also presented as an immunomodulatory effect via mutual reaction with CD4^+^CD25^+^ Tregs. Interestingly, CD4^+^CD25^+^ Tregs may inhibit the IFN-γ production via activated γδ T cells. Moreover, the activated Vγ9Vδ2 subpopulation of T cells may downregulate the expansion of Tregs (CD4^+^CD25^+^Foxp3^+^) induced by IL-2. These findings suggest that CD4^+^CD25^+^ Tregs and γδ T cells interact, both playing a significant role in the pathogenesis of a multitude of autoimmune diseases [57,58,59]. Figure 2 shows what is currently known about autoimmune diseases associated with γδ T lymphocytes.

## 5. γδ T Lymphocytes in Endometriosis

According to some studies, endometriosis (EMS)—a chronic disease of the female reproductive system characterized by the presence and growth of endometrium-like tissue beyond its normal location in the uterus—has some features of an autoimmune disease. It is manifested by tissue damage and the production of autoantibodies (against the endometrium, histones, ovaries, and phospholipids), and may be associated with other autoimmune diseases [60,61,62,63]. In this progressive disease, the immune system plays an important role based on a cellular type of immune response, e.g., NK cells, monocytes/macrophages, or T lymphocytes [5,64,65]. It is worth noting that many factors are involved in the immunology and pathogenesis of endometriosis. The research describes the participation of IL-17, which causes an increase in the secretion of IL-8 from endometriotic stromal cells, stimulating their proliferation, and an increase in neutrophil migration. IL-17A expression through endometrial cell stimulation leads to the accumulation of neutrophils, resulting in continuous inflammation—a distinctive feature of endometriosis [65]. Th17 lymphocytes, neutrophils, NK cells, and γδ T lymphocytes are responsible for the secretion of IL-17 [5,64].

Th17 cells play a key role in the immune system. They are responsible for triggering inflammation, inducing the synthesis of pro-inflammatory cytokines and helping in the recruitment of neutrophils [64]. Th17 cells also produce chemokines (e.g., CXCL1, CXCL5, CCL2, CCL7), which are involved in the recruitment of neutrophils at the inflammation site. Extensive research exists showing that neutrophils play an important role in most inflammatory diseases, including endometriosis. There is evidence that patients with EMS have increased neutrophil levels in the peritoneal fluid (PF). Neutrophil-produced pro-inflammatory factors such as IL-8, VEGF, and chemokine C-X-C motif ligand 10 (CXCL10) may contribute to the progression of EMS [64]. Studies have shown that a decreased ratio between Th17 and CD4^+^ T lymphocytes also contributes to the progression of EMS, which leads to excessive ectopic proliferation of endometrial tissue [64]. The influence of IL-17 on endometrial cells and its role in increasing TNFα production may contribute to the acceleration of endometrial lesions in women with EMS. It is worth mentioning that in patients suffering from EMS in their PF, an increased number of Th17 cells causes disease progression to a severe stage when coupled with a higher concentration of IL-8 and a lower concentration of IL-12 [64].

It should be stressed that only a few reports have shown the presence of γδ T cells in endometriosis [4,5]. Table 1. shows what is currently known about γδ T lymphocytes in patients suffering from EMS. However, since insufficient information is known about γδ T cells, further research is warranted to fully understand their role in endometriosis.

## 6. γδ T-Cell-Based Immunotherapy and Its Limitations

Immunotherapy is of considerable interest to scientists as a new field in the fight against cancer. It has the clear advantage of deploying the patient’s immune system against the cancer that the patient is struggling with [66,67]. For a long time, immunotherapy relied on αβ T lymphocytes. However, their strong dependence on the histocompatibility system had a profound effect on immunotherapeutic outcomes [66,67,68]. Hence, immunotherapy based on γδ T lymphocytes has brought considerable hope due to the absence of CD4/CD8 antigen expression and independence from the MHC system, with the lymphocytes being considered a bridge between the innate and adaptive immune systems [66,67,68,69].

One of the most important functions of γδ T cells is their cytotoxicity through the production of numerous chemokines and cytokines, including TNFα [66]. Moreover, these cells are involved in the regulation of both immune and non-immune cells. The most important cytokine produced by γδ T lymphocytes is INF-γ, which shows antitumor activity [66]. γδ T cells are also described as professional antigen-presenting cells (APCs) that stimulate antigen-specific αβ T-cell responses [10]. Brandes et al. showed that Vγ9Vδ2 T cells were more efficient in presenting antigens and exhibited a 100-fold higher proliferative activity in comparison to αβ T lymphocytes or monocytes [70].

Furthermore, these lymphocytes may participate in the regulation of the immune response due to their interaction with other immune cells. This illustrates cells bearing features for innate and adaptive immunity. Their unique immune-modulating functions and tropism to the TME make them an attractive target for cancer therapy [10,66,68,69]. γδ T lymphocytes provide the basis of immunotherapy in the treatment of, among others, renal cell carcinoma, and lung and breast cancer. They offer the advantage of activating a response to the tumor and not to healthy cells. The use of activators for γδ T lymphocytes, i.e., phosphoantigens or compounds (zoledronate) results in their effective activation [66,68]. Despite the numerous advantages associated with γδ T cell immunotherapy, there are also some limitations. In addition to other factors, they are also attributable to the dual role of lymphocytes, which contribute to undesirable effects through the production of pro-inflammatory cytokines. According to Zou et al. in 2017 [66], the continued use of phosphoantigen activators leads to the anesthesia of effector cells, and the targeting of γδ T cells and antibodies as antitumor agents could represent a significant leap forward in the field of immunotherapy [66].

## 7. γδ T Cells—Clinical Trials

Information on clinical trials involving the use of gamma-delta T cells in gynecological diseases is rudimentary and scarce. Only one such study, involving ovarian cancer, has been described so far. In 2012, a French team of researchers planned a study for patients with confirmed epithelial ovarian cancer and administration of carboplatin and/or Taxol chemotherapy. Unfortunately, the study was eventually terminated [71].

We can also find information about a project named “Immunotherapy of Epithelial Ovarian Cancer using Autologous Gamma Delta T-cells” planned by King’s College London. The research will be carried out on immunodeficient mice. The first step consists in delivering bisphosphonates to ovarian cancer cells by injection, followed by injection of γδ T-cells. If these studies prove promising, the next step will be to develop a strategy for women with ovarian cancer [72].

In 2016, clinical studies on the safety and efficacy of γδ T cells against breast cancer were completed. These studies included 40 female patients aged between 18 and 75 years who had been diagnosed with stage II, III, or IV breast tumors. The primary finding was that the tumor size reduced within a time frame of up to one year [73]. The ongoing clinical trials have been focused on the use of γδ T cells in the general therapy of solid tumors and, ultimately, in glioblastoma. The first trial, now in phase II, includes 60 male and female patients aged between 18 and 75 years. Depending on the patient’s health status, these trials can be combined with radiotherapy, chemotherapy, immune checkpoint inhibitors, or targeted therapy. The trial is scheduled to end in December 2024 [74].

The second ongoing trial involves patients with newly diagnosed glioblastoma, including 12 adult patients with histologically confirmed glioblastoma multiforme. The treatment received as part of the trial complements standard therapies such as radiotherapy and chemotherapy with temozolomide. The main aim of the trial is to determine the safety and toxicity of intracranially infused γδ T cells. The disease progression duration and average survival times are also assessed. The trial is currently in phase I with completion scheduled for January 2025 [75].

There is some currently conducted research into allogeneic therapy with γδ T cells in the treatment of solid tumors (Identifier: NCT04765462). These studies are in phase I/II and their task is to determine the feasibility, efficacy, and safety of therapy with the use of γδ T cells. The current research status is Recruiting. The study is funded by the Chinese PLA General Hospital and is expected to involve 60 patients, both women and men, aged between 18 and 75 years with histological confirmation of malignant solid tumors in both initial and metastatic stages [76].

## 8. The Effect of Conventional Therapies on the Function of γδ T Lymphocytes 

γδ T cells are important players in the immunological surveillance of tumors, both in vitro and in vivo. Many studies use chemical compounds whose task is to affect the functioning of γδ T cells and enhance their proliferation [8,68,77]. Experimental studies and several clinical trials based on ex vivo expanded γδ T cells confirmed that their adoptive transfer is safe and feasible. The use of activators for γδ T lymphocytes, i.e., phosphoantigens or such compounds as zoledronic acid (ZOL), results in effective activation of Vγ9Vδ2 T lymphocytes through upregulated levels of endogenous PAgs [66,68]. In most γδ T-cell clinical trials, objective responses were observed but the rates of complete remissions were low and the long-term disease-free survival data were unsatisfactory [10,69,78]. Taking into account this observation, there is a need for approaches that would more effectively enhance the antitumor efficacy of these cells [77,79].

Recent studies have demonstrated that some drugs or therapies can elicit tumor cell death and modulate γδ T cell activity [78,80,81]. It has been shown that chemotherapy and radiotherapy increase the cytotoxicity of γδ T lymphocytes against malignant cells [77,78,82]. For example, pretreatment of cancer cells with conventional chemotherapeutics, e.g., gemcitabine [78], cisplatin, 5-Fluorouracil, or doxorubicin, sensitize tumor cells to being killed by γδ T cells [77,79,80]. After exposure to cytotoxic treatment, neoplastic cells upregulate the expression of MICA and MICB, which increases the anti-tumor activity of γδ T cells [83]. Another in vitro study concluded that combined chemotherapy (carboplatin with γδ T cells) had shown significant cytotoxicity against bladder carcinoma cells compared with chemotherapy alone [84].

It has also been demonstrated that chemotherapy induces a rapid and prominent expansion of IL-17-producing Vγ4^+^ and Vγ6^+^ T cells and γδ T17 cells which augment the accumulation of cytotoxic T cells (Tc1 CTLs) within the tumor bed [85]. The authors observed reduced efficacy of chemotherapy in mice without Vγ4/γ6 T cell subsets or δ- in T cell receptors. Although γδ T cells were able to produce IL-17 and IL-22, the absence of IL-17–IL-17R reduced tumor-specific T cell responses elicited by chemotherapy-induced tumor cell death [85].

Joalland N. et al. [86] evaluated the efficiency of conventional therapies and adoptive transfers of allogeneic human Vγ9Vδ2 T cells in a preclinical model of human epithelial OC xenografts. The authors observed no disease symptoms (e.g., weight loss, production of ascites) up to 6 months after implantation of human OC cells into the ovary of immunodeficient NSG (NOD.Cg-Prkdcscid Il2rgtm1Wjl/SzJ) mice. In line with the results of a biological assessment, there was no evidence of signs of peritoneal carcinosis, and both immunochemistry and necropsy analyses revealed the absence of a suboptimal growth of cancer cells. The studies showed that neoplastic cells, originating from either primary OC or ovarian cancer cell lines, were targeted by allogeneic human Vγ9Vδ2 T lymphocytes in vitro upon ZOL treatment [86].

In their in vitro studies, Beatson et al. [79] observed that sublethal exposure to the antileukemic drug, Ara-C, rendered leukemic cells more susceptible to being killed by γδ T cells expanded in a medium with IL-2 and TGF-β. However, the sensitization by Ara-C neither increased IFN-γ release nor upregulated NKG2D ligands on leukemic cells. The authors also tested antileukemic activity in vivo in a Jurkat xenograft model. They detected that the combination of ZOL^+^Ara-C boosted the response of γδ T cells, improving both disease control and survival. On the contrary, neither ZOL^+^Ara-C alone nor combined with γδ T cells was effective. Further studies in KG1 leukemia xenografts showed that, in mice sensitized with ZOL^+^Ara-C, γδ T cells delayed disease progression and prolonged survival. In summary, the authors observed strong therapeutic activity of these cells in leukemic model systems in the described conditions [79].

It has been observed that the use of ibrutinib (a tyrosine kinase inhibitor) in the treatment of chronic lymphocytic leukemia, whose task is to support the effectiveness of endogenous gamma-delta T cells, partially reverses the tolerance of lymphocytes to patient’s cancer cells in vitro [68].

In addition, several drugs, including the DNA methyltransferase inhibitor decitabine and the histone deacetylase inhibitor-valproic acid (VPA) have been shown to epigenetically modify gene expression at the level of DNA methylation and histone modification [81]. Decitabine was reported to upregulate the expression of NKG2D ligands in tumor cells [77,87]. VPA was shown to synergize with ZOL in enhancing γδ T cell cytotoxicity at the level of PAg synthesis. It also affected the interaction between γδ T cells and tumor cells at the level of the NKG2D receptor/ligand axis [81].

Overall, combination therapies involving conventional chemotherapeutic drugs and γδ T cell immunotherapies offer interesting perspectives for the treatment of cancer [77].

## 9. Conclusions

Immunotherapy with γδ T cells is known to bear certain limitations and disadvantages. This is due to the dual role played by γδ T cells. On the one hand, they exhibit strong anti-cancer effects. On the other, these cells can also show pro-tumor activity. It depends on the nature of the tumor microenvironment [11,66,68,69]. There are also some technical and functional limitations in using γδ T cells in cancer therapy. Technical problems could be related to the hyporesponsiveness of γδ T cells in some patients, or to activation-induced γδ T cell anergy. Functional limitations could be related to the ability of in vivo or in vitro-expanded γδ T cells to reach and infiltrate tumors and overcome the immunosuppressive TME [32]. To overcome this limitation and improve γδ T-cell-based immunotherapy, several new immunotherapeutic approaches have been studied involving immunological checkpoint inhibitors, bispecific antibodies, chemotherapy, liposomes, and chimeric antigen receptor-T cells (CAR-T) [88]. Considering the heterogeneity of γδ T cells, it seems critical to understand their role and interactions with different types of innate and adaptive immune cells in the TME. It is necessary to better describe human γδ T cell subsets in specific TMEs of particular cancers.

## Figures and Tables

**Table 1:** The role of Vδ T cells in the pathogenesis of endometriosis (EMS).

Vδ T in EMS	Implications of Vδ T in EMS
The number of circulating Vδ1 T cells was significantly reduced in women suffering from endometriosis [4]. An increased proportion of Vδ1 T cells in the eutopic and ectopic layers of the endometrium in patients with EMS compared to the controls [4].	**Vδ1 T cells may be involved in:** the dysfunction of the eutopic layer of the endometrium the promotion and maintenance of inflammation the formation of endometrial lesions [5].
Vδ T cells are an important source of IL-17 [63]	**IL-17A:** increases the secretion of IL-8 from endometriotic stromal cells stimulates the proliferation of endometriotic stromal cells increases migration of neutrophils which stimulates a continuous inflammatory state [63].

## Data Availability

Not applicable.
